# Medical Items

**Published:** 1893-12

**Authors:** 


### *MEDICAL ITEMS.*

WE are indebted to Messrs. J. H. Chambers & Co., St. Louis, for the last issue of their *Visiting List.* We take pleasure in recommending it to those purchasing lists for 1894. Price, 75 cents.

WE have received a copy of Blakiston's *Visiting List* for 1894. It has enjoyed a popularity with the profession for forty-three years, and is certainly one of the most convenient and serviceable. Price, one dollar. P. Blakiston, Son & Co., 1012 Walnut St., Philadelphia.

WE acknowledge with pleasure the receipt of the first issue of the *Tri-State Medical Journal,* published at Keokuk, Iowa, and edited by Dr. Jas. Mores Ball. It will be the official journal of the Tri-State Medical Society of Iowa, Illinois and Missouri. The first number is made up of good matter, well arranged, and gives promise of future success.

DR. CHARLES W. EARLE, of Chicago, died November 19th. Dr. Earle was one of the most prominent physicians of Chicago, and at the time of his death was President of the Chicago Medical Society and Professor of Diseases of Children in the College of Physicians and Surgeons.

WE regret to learn of the death of Dr. John M. Keating, the well known author of Keating's Dictionary, and editor of the Encyclopedia of the Diseases of Children. Dr. Keating was formerly a resident of Philadelphia, but moved to Colorado some years ago on account of his health. He had led a very active life in his profession and had made many contributions to medical literature.

JUDGE RICHARD CLARK, of the Atlanta criminal court, has abolished the custom of kissing the book, in the interest of health. The advocates of a rigid hygiene long ago called attention to the possible danger in the practice of touching one's lips to the begrimed volume that had been kissed before by promiscuous thousands, and we cannot but regard Judge Clark's decision as a wise one. It is at least another step in the interest of cleanliness.

---

FOR the past five years the meetings of the Tri-State Medical Society of Georgia, Alabama and Tennessee have been held in Chattanooga. Hereafter they will divide their annual sessions between the three States, and the next meeting will be held in Atlanta, October, 1894. Following are the officers for the ensuing year: President, J. B. S. Holmes, Georgia; Vice-Presidents, Jas. A. Goggans, Alabama; D. H. Howell, Georgia, and T. Hilliard Wood, Tennessee; Secretary, Frank Trester Smith, Chattanooga, Tenn.

---

DR. JUDSON DALAND, of Philadelphia, the editor of our excellent contemporary, the *International Medical Magazine*, seems to be something of a swimmer. On a late visit to Italy and Sicily it occurred to him to swim the celebrated whirlpool between Scylla and Charybdis. The distance is about six miles, and no one had ever attempted to swim it. Dr. Daland started from the Sicilan side at 4:10 P. M., passed the rock of Scylla and landed on the Italian shore at 6:30 P. M. The swim was made without rest or stimulants. The doctor "returned to Messina in good condition and that same evening went to the opera."

